# Intra-Aortic Balloon Pump among Shockable Out-of-Hospital Cardiac Arrest Patients: A Propensity-Weighted Analysis in a Multicenter, Nationwide Observational Study in Japan (The JAAM-OHCA Registry)

**DOI:** 10.3390/jcm12185945

**Published:** 2023-09-13

**Authors:** Satoshi Yoshimura, Takeyuki Kiguchi, Taro Irisawa, Tomoki Yamada, Kazuhisa Yoshiya, Changhwi Park, Tetsuro Nishimura, Takuya Ishibe, Hitoshi Kobata, Masafumi Kishimoto, Sung-Ho Kim, Yusuke Ito, Taku Sogabe, Takaya Morooka, Haruko Sakamoto, Keitaro Suzuki, Atsunori Onoe, Tasuku Matsuyama, Satoshi Matsui, Norihiro Nishioka, Yohei Okada, Yuto Makino, Shunsuke Kimata, Shunsuke Kawai, Ling Zha, Kosuke Kiyohara, Tetsuhisa Kitamura, Taku Iwami

**Affiliations:** 1 Department of Preventive Services, Kyoto University School of Public Health, Kyoto 606-8317, Japan; yoshimura.satoshi.s34@kyoto-u.jp (S.Y.); nishioka.norihiro.26x@st.kyoto-u.ac.jp (N.N.); okadayohei1127@yahoo.co.jp (Y.O.); makino.yuto.83n@st.kyoto-u.ac.jp (Y.M.); shunchenco@yahoo.co.jp (S.K.); kawai_shunsuke@yahoo.co.jp (S.K.); 2 Critical Care and Trauma Center, Osaka General Medical Center, Osaka 558-8558, Japan; take_yuki888@yahoo.co.jp; 3 Department of Traumatology and Acute Critical Medicine, Graduate School of Medicine, Osaka University, Suita 565-0871, Japan; taroirisawa@yahoo.co.jp; 4 Emergency and Critical Care Medical Center, Osaka Police Hospital, Osaka 543-0035, Japan; tomoki27@hp-emerg.med.osaka-u.ac.jp; 5 Department of Emergency and Critical Care Medicine, Kansai Medical University Takii Hospital, Moriguchi 570-8507, Japan; kyoshiya@msn.com; 6 Department of Emergency Medicine, Tane General Hospital, Osaka 550-0025, Japan; parkchanghwi@gmail.com; 7 Department of Traumatology and Critical Care Medicine, Osaka Metropolitan University, Osaka 545-8585, Japan; tetusayo2004@mail.goo.ne.jp; 8 Department of Emergency and Critical Care Medicine, Kindai University School of Medicine, Osaka-Sayama 589-8511, Japan; t-ishibe@med.kindai.ac.jp; 9 Osaka Mishima Emergency Critical Care Center, Takatsuki 569-1124, Japan; kobata106@osaka-mishima.jp; 10 Osaka Prefectural Nakakawachi Medical Center of Acute Medicine, Higashi-Osaka 578-0947, Japan; mdmkishi@db3.so-net.ne.jp; 11 Senshu Trauma and Critical Care Center, Izumisano 598-8577, Japan; a06m038@yahoo.co.jp; 12 Senri Critical Care Medical Center, Saiseikai Senri Hospital, Suita 565-0862, Japan; yuusukeito@hotmail.com; 13 Traumatology and Critical Care Medical Center, National Hospital Organization Osaka National Hospital, Osaka 540-0006, Japan; sogataku@gmail.com; 14 Emergency and Critical Care Medical Center, Osaka City General Hospital, Osaka 534-0021, Japan; bubuzuke0319@gmail.com; 15 Department of Pediatrics, Osaka Red Cross Hospital, Osaka 543-8555, Japan; sakamoto@osaka-med.jrc.or.jp; 16 Emergency and Critical Care Medical Center, Kishiwada Tokushukai Hospital, Kishiwada 596-8522, Japan; metal0129@gmail.com; 17 Department of Emergency and Critical Care Medicine, Kansai Medical University, Hirakata 573-1010, Japan; ogami_0424@yahoo.co.jp; 18 Department of Emergency Medicine, Kyoto Prefectural University of Medicine, Kyoto 602-8566, Japan; kame0413oka.jin@gmail.com; 19 Division of Environmental Medicine and Population Sciences, Department of Social and Environmental Medicine, Graduate School of Medicine, Osaka University, Suita 565-0871, Japan; matsui0620@gmail.com (S.M.); sarin@envi.med.osaka-u.ac.jp (L.Z.); lucky_unatan@yahoo.co.jp (T.K.); 20 Health Services and Systems Research, Duke-NUS Medical School, Singapore 169857, Singapore; 21 Department of Food Science, Otsuma Women’s University, Tokyo 102-8357, Japan; kiyosuke0817@hotmail.com

**Keywords:** out-of-hospital cardiac arrest, intra-aortic balloon pump, cardiopulmonary resuscitation

## Abstract

Background: The effectiveness of IABP for shockable out-of-hospital cardiac arrest (OHCA) has not been extensively investigated. This study aimed to investigate whether the use of an intra-aortic balloon pump (IABP) for non-traumatic shockable OHCA patients was associated with favorable neurological outcomes. Methods: From the Japanese Association for Acute Medicine Out-of-Hospital Cardiac Arrest registry, a nationwide multicenter prospective registry, we enrolled adult patients with non-traumatic and shockable OHCA for whom resuscitation was attempted, and who were transported to participating hospitals between 2014 and 2019. The primary outcome was 1-month survival with favorable neurological outcomes after OHCA. After adopting the propensity score (PS) inverse probability of weighting (IPW), we evaluated the association between IABP and favorable neurological outcomes. Results: Of 57,754 patients in the database, we included a total of 2738 adult non-traumatic shockable patients. In the original cohort, the primary outcome was lower in the IABP group (OR with 95% confidence intervals (CIs)), 0.57 (0.48–0.68), whereas, in the IPW cohort, it was not different between patients with and without IABP (OR, 1.18; 95% CI, 0.91–1.53). Conclusion: In adult patients with non-traumatic shockable OHCA, IABP use was not associated with 1-month survival with favorable neurological outcomes.

## 1. Introduction

Out-of-hospital cardiac arrest (OHCA) is a significant public health issue in the industrialized countries, with about 120,000 events occurring annually in Japan and 360,000 in the United States [1,2]. OHCA with shockable rhythms, such as ventricular fibrillation (VF) and pulseless ventricular tachycardia (pVT), is a crucial aspect of the resuscitation strategy since these arrhythmias are treatable with defibrillation and are more likely to have cardiogenic origin [3].

The significance of post-cardiac arrest care for OHCA patients who have achieved cardiopulmonary resuscitation has been recently emphasized [4,5]. In the context of post-cardiac arrest syndrome, patients may experience multiorgan failure and cerebral dysfunction due to low cardiac output and high systemic vascular resistance [4,5]. In this clinical setting, IABP offers modest improvements in cardiac output and coronary perfusion, reduces afterload, maintains organ perfusion, and has been utilized as percutaneous mechanical circulatory support devices (pMCS) for OHCA patients who have achieved a return of spontaneous circulation (ROSC) in the post-cardiac arrest phase [6]. However, in a randomized trial conducted among non-OHCA patients with cardiogenic shock caused by acute myocardial infarction, we did not observe any benefits of IABP use in terms of 30-day and 1-year mortality rates [7]. Additionally, several observational studies failed to demonstrate any benefit of IABP use on survival outcomes among adult patients with OHCA [8,9]. Due to the limited number of patients, the impact of IABPs on favorable neurological outcomes in cardiac arrest cases with shockable rhythms could not be adequately evaluated in these studies. Consequently, the question of whether the utilization of IABP improves outcomes for OHCA patients with shockable rhythms who have achieved ROSC remains a subject of controversy.

In this study, we evaluated whether the use of IABP for non-traumatic and shockable OHCA patients after ROSC was associated with favorable neurological outcomes after ROSC based on propensity score (PS) weighting, using inverse probability of weighting (IPW) for average treatment effect among treated patients, and using data from the nationwide multicenter OHCA registry in Japan.

## 2. Materials and Methods

This study adhered to the guidelines outlined in the Strengthening the Reporting of Observational Studies in Epidemiology (STROBE) statement, which provides a framework for reporting observational studies [10]. The research protocol received approval from the Ethics Committee of the Kyoto University Graduate School of Medicine (R1045) as well as the participating hospitals. Given the nature of the observational study, the need for individual patient consent was waived.

### 2.1. Study Design and Setting

The data for this observational study were obtained from the Japanese Association for Acute Medicine Out-of-Hospital Cardiac Arrest (JAAM-OHCA) registry. The JAAM-OHCA registry is a comprehensive and prospective database that encompasses OHCA patients who were transferred to various participating hospitals across the nation. A previous publication has provided a thorough description of the registry’s specifics [11]. To provide a comprehensive dataset, the JAAM-OHCA registry incorporates pre-hospital information gathered by emergency medical services, following the standardized Utstein-style template. Additionally, in-hospital information, such as treatment, outcomes, and clinical data, is reported by physicians or clinical data managers. As of December 2019, a total of 83 hospitals in Japan, including tertiary emergency centers and university hospitals, actively participated in the registry. The enrollment period for the available data spans from June 2014 to December 2019, encompassing a total of 57,754 patients.

### 2.2. Study Patients

For this study, we included all consecutive OHCA patients aged 18 years or older who underwent resuscitation attempts and were subsequently transferred to the participating institutions. The enrollment period for these eligible patients spanned from June 2014 to December 2019. OHCA patients who did not receive cardiopulmonary resuscitation from physicians upon arrival at the hospital or who had disagreements regarding their inclusion in the registry were excluded from the analysis. Among these patients, those with a shockable rhythm, specifically those with ventricular fibrillation or pulseless ventricular tachycardia as the first documented rhythm at the scene, were eligible for analysis. The requirement for obtaining individual informed consent for registration was waived.

### 2.3. Emergency Medical Services in Japan

A previous publication outlined the emergency medical services (EMS) system in Japan [11]. To provide a brief overview, Japan utilizes the emergency telephone number “119” accessible nationwide. When a 119 call is received, the emergency dispatch center dispatches the nearest available ambulance to the location. The emergency services are available 24 h a day. Each ambulance is staffed with a three-person unit trained in providing life support. Specially trained emergency life-saving technicians are authorized to perform endotracheal intubation and administration of epinephrine to OHCA patients. All emergency medical service providers adhere to the Japanese cardiopulmonary resuscitation guidelines for performing cardiopulmonary resuscitation [12]. For the collection of pre-hospital resuscitation data, the All-Japan Utstein Registry of the Fire and Disaster Management Agency of Japan was utilized. The data collection process followed a form based on the Utstein-style international guidelines for reporting OHCA, ensuring consistency and adherence to standardized reporting protocols [13].

### 2.4. Data Collection and Quality Control

The data collection and quality control process for the registry has been documented in another publication [11]. Collaborating with the attending physician responsible for the patient, each emergency medical service completed the data form for data collection. For in-hospital data collection and quality control, the JAAM-OHCA registry gathers comprehensive data on OHCA patients following their arrival at the hospital, as described in a previous study [11]. In this registry, anonymized data were entered into a web sheet by a physician or medical staff member working with the attending physician. Pre-hospital and in-hospital data were uploaded into the registry system and subjected to logical checks performed by a computer system. Subsequently, a working group comprising experts in emergency medicine and clinical epidemiology verified the data to ensure its accuracy and reliability.

### 2.5. Outcome Measures

The primary outcome in this study was 1-month survival with favorable neurological outcomes after OHCA. The secondary outcome was 1-month survival. We defined a favorable neurological outcome as a cerebral performance category score of 1 or 2.

### 2.6. Sample Size Estimation and Missing Value

For this study, we utilized the entirety of the data accessible within the aforementioned registry, which is recognized as the largest and most comprehensive OHCA registry in Japan. The missing values were imputed using the MissForest method [14].

### 2.7. Statistical Analysis

Pre- and in-hospital information and outcomes were compared between patients with and without IABP. For reducing the potential confounding effects in the comparison between IABP and non-IABP use, we performed propensity score (PS) weighting using inverse probability of weighting (IPW) for average treatment effect as a primary analysis.

Firstly, we estimated a PS using a logistic regression model that adjusted for the following 14 variables: year (2014–2019), sex (male or female), age (continuous value), witness status (no, yes), bystander cardiopulmonary resuscitation (no, yes), adrenaline administration at pre-hospital (no, yes), tracheal intubation at pre-hospital (no, yes), duration from call to hospital arrival (continuous value), first documented rhythm after hospital arrival (shockable, non-shockable, presence of pulse), adrenaline administration in-hospital (no, yes), coronary angiography (no, yes), target temperature management (no, yes), extracorporeal membrane oxygenation (no, yes), and cause of arrest (cardiac, noncardiac). We chose these variables, which potentially affect the probability of treatment assignment, based on clinical knowledge and previous studies [8]. We performed receiver operating characteristic curve analysis with an area under the curve of PS for predicting IABP use in patients with OHCA. For the primary analysis, treatment effect estimation was performed using IPW, calculating the average treatment effect on the treated patients. For the secondary analysis, one-to-one pair matching between the IABP and non-IABP groups was performed. For the matching, we used nearest neighbor matching without replacement with calipers of width equal to 0.2 of the standard deviation of the logit of PS. We checked the standardized mean difference before and after IPW and matching to measure covariate balance. When the standardized mean difference was <0.25, there was a negligible imbalance between the two groups [15]. In both IPW and PS-matched analyses, we investigated the association between the IABP group and non-IABP group and favorable neurological outcomes using univariable logistic regression analyses. We calculated the crude odds ratio (OR) and 95% confidence intervals (CIs) based on these analyses. All p values were two-sided, and statistical significance was set at *p* < 0.05. All statistical analyses were performed using STATA version 16.0 SE software (Stata Corp LP, College Station, TX, USA) and R studio (Version 1.2.5033). In addition, we performed subgroup analysis by the cause of arrest, witness status, first documented rhythm after hospital arrival, and reperfusion therapy using the univariable and multivariable logistic regression analyses.

In this study, patients or the public were not involved in the design, conduct, reporting, or dissemination plans of our research.

## 3. Results

**Figure 1:**
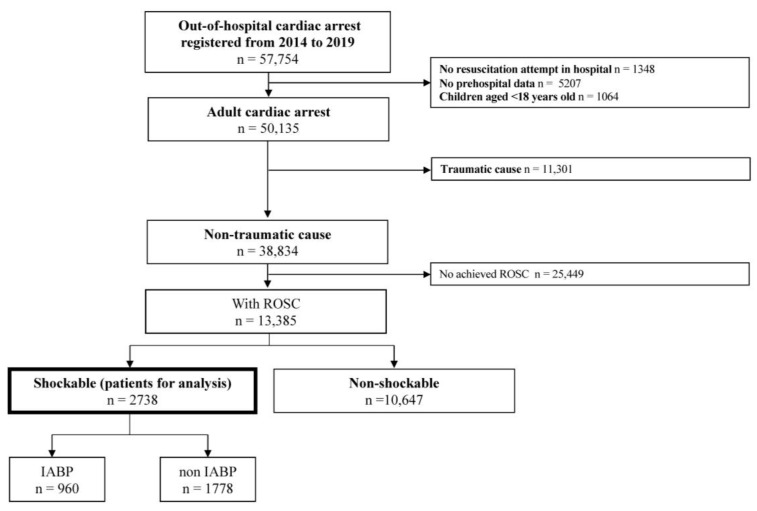
Patient flow. IABP, intra-aortic balloon pump; ROSC, return of spontaneous circulation.

Figure 1 shows an overview of the study population. A total of 57,754 OHCA patients were documented between January 2014 and December 2019. After excluding 1348 patients who were not resuscitated by physicians on hospital arrival, 5207 patients without pre-hospital data, and 1064 pediatric patients, a total of 2738 adult patients were eligible for analysis.

Table 1 and Table 2 show the baseline characteristics of 2738 patients who achieved ROSC after non-traumatic OHCA with shockable rhythm by IABP use in the original cohort (Table 1) and PS-matched cohort (Table 2). The standard mean differences in the original, IPW are shown in Table 1, and those in the PS-matched cohort are shown in Table 2. In the original cohort, patients in the IABP group were more likely to be younger; male; administered adrenaline both out- and in-hospital; to have shockable rhythm, to undergo coronary angiography, percutaneous coronary intervention, target temperature management, and extracorporeal membrane oxygenation; and to have a cardiac cause. In the PS-matched cohort, 468 patients in each group were selected from the original cohort. The area under the receiver operating curve of PS with 95% confidence interval (CI) was 0.879 (0.865–0.892). The covariates between the score-matched and IPW groups were well-balanced.

Table 3 shows the crude ORs in the original, IPW, and PS-matched cohorts. In the original cohort, 1-month survival with a favorable neurological outcome was lower in the IABP group (OR, 0.57; 95% CIs, 0.48–0.68). However, in the IPW cohort, 1-month survival with favorable neurological outcomes was not different between patients with and without IABP (OR, 1.18; 95% CI, 0.91–1.53). This result was consistent in the secondary analysis among the PS-matched patients (OR, 0.97; 95% CI, 0.75–1.25).

Table 4 shows the results of the subgroup analyses. In the subgroup analysis on the cause of arrest, witness status, first documented rhythm after hospital arrival including the presence of pulse and the reperfusion therapy, 1-month survival with a favorable neurological outcome, and 1-month survival were not different between the patients with and without IABP.

## 4. Discussion

### 4.1. Summary

In this study, IABP use for adult patients who achieved ROSC after non-traumatic OHCA with shockable rhythm did not improve 1-month survival with favorable neurological outcome in the IPW analysis for estimating the average treatment effect among treated patients. These results were consistent with those of the secondary analysis in the PS-matched cohort.

### 4.2. Comparison with Previous Studies

This study examined the association between IABP use and neurological outcomes in OHCA patients using a Japanese nationwide multicenter registry. Previously, some RCTs revealed that IABP did not improve 30-day mortality in acute myocardial infarction and shock cases [7,16], but it was unclear whether IABP improved the clinically relevant outcomes among cardiac arrest patients. Our previous report using PS-matching also demonstrated that IABP was not associated with a better neurological outcome in patients with non-traumatic OHCA [8]; however, this study may have lacked power because of the sample size (N = 316 in the matched cohort). In addition, this study might have unmeasured bias due to the heterogeneity of the patients’ background because the study included both shockable and non-shockable patients. In this present study, we used a nationwide registry to investigate the effectiveness of IABP in the shockable group, which is considered an active resuscitation target in the current guideline and is frequently cardiogenic, for which IABP would theoretically be effective [3]. However, IABP did not improve favorable neurological outcomes. Our subgroup analysis investigating the effect of IABP by age, presence of witnesses, initial waveform at hospital arrival, and presence of PCI also revealed no difference between patients with and without IABP.

### 4.3. Possible Explanation and Implications

There are several possible explanations for these results. First, because IABP provides less hemodynamic support than other pMCSs (i.e., cardiac flow of IABP, Impella, and Tandem Heart are 0.3–0.5 L/min, 1.0–5.0 L/min, and 2.5–5.0 L/min, respectively), the IABP might not sufficiently support the circulation system among patients with post-cardiac arrest syndrome [17]. Previous studies in patients with acute myocardial infarction and shock have reported that IABP did not improve cardiac output, left ventricular stroke work index, or systemic vascular resistance [7,16]. IABP might become effective if combined with other devices, such as extracorporeal life support [18]. There is no mention of IABP use in the latest resuscitation guidelines [19,20], and it is not recommended by the myocardial infarction guidelines [21,22]. This study supports these recommendations, and we believe that routine use of IABP is not recommended, even in cases of OHCA. However, future studies are needed to examine the target group of OHCA for which IABP is effective. Additionally, the integration of IABP with other interventions, such as venoarterial extracorporeal membrane oxygenation (VA-ECMO), is important. This combination therapy aims to optimize left ventricular unloading facilitated by IABP and ameliorate the north–south syndrome [20]. Additionally, studies investigating the effectiveness of pMCS other than IABP on the neurological prognosis of OHCA are mandated. Particularly, in terms of the target group of the IABP administration, considering patient comorbidities and clinical factors is crucial for long-term prognosis, such as age, history of diabetes mellitus, and renal failure are predominant predictors of OHCA with coronary artery disease [23,24]. These factors and other co-existing factors, such as congestive heart failure and cancer, were included as potential explanatory variables for the analysis. Although these factors were not evaluated in our registry, we need to consider them in future studies.

### 4.4. Limitation

This study had several limitations. First, measured confounders were adjusted for in the PS, but unmeasured confounders (e.g., indication bias) may not have been adjusted for. In addition, owing to the nature of the registry, details of cardiogenic disease (e.g., mechanical complications) were not measured and were unknown. Furthermore, we did not measure cardiovascular events during the course of the study (e.g., worsening heart failure, fatal arrhythmia during hospitalization, or recurrent acute myocardial infarction during hospitalization) or as long-term prognosis, and it is unclear whether the use of IABP is associated with these outcomes. The transportability of our results to countries other than Japan is another limitation. This is due to the fact that emergency medical services in Japan are not authorized to terminate cardiopulmonary resuscitation (CPR) without a physician present at the scene. Consequently, our current findings may not be applicable to more selective populations where only patients with ROSC are transported to hospitals. Finally, the results of this present study cannot be applied to extracorporeal cardiopulmonary resuscitation cases because the inclusion criteria for extracorporeal cardiopulmonary resuscitation cases are different from those of this present study.

## 5. Conclusions

The current PS weighting analysis demonstrated that IABP use did not improve 1-month survival with favorable neurological outcomes among adult patients with ROSC after OHCA with shockable rhythm.

## 6. Patents

This study was supported by a scientific research grant from the JSPS KAKENHI of Japan (22H03313 to Iwami and 22K09139 to Kitamura). YO received a research grant from the ZOLL foundation and an overseas scholarship from the FUKUDA Foundation for Medical Technology and the International Medical Research Foundation. These organizations had no role in conducting this research. All other authors have reported that they have no relationships relevant to the contents of this paper.

## Figures and Tables

**Table 1:** Characteristics of patients in original cohort.

	Original Cohort				
	With IABP	Without IABP	Missing (%)	SMD	SMD (IPW)
	(N = 960)	(N = 1778)			
Year, *n*(%)				0.051	0.015
2014	74 (7.7)	134 (7.5)	0		
2015	143 (14.9)	297 (16.7)	0		
2016	166 (17.3)	317 (17.8)	0		
2017	177 (18.4)	343 (19.3)	0		
2018	210 (21.9)	358 (20.1)	0		
2019	190 (19.8)	329 (18.5)	0		
Age, years, median (IQR)	63.00 [52.00, 71.00]	66.00 [54.00, 76.00]	0	0.215	0.04
Men, *n* (%)	816 (85.0)	1373 (77.2)	0	0.2	0.007
Witness status, *n* (%)	395/100 (79.8/20.2)	384 (73.0)	0	0.021	0.039
Bystander CPR	538 (56.0)	1012 (56.9)	0	0.018	0.009
Adrenaline administration at pre-hospital, *n* (%)	323 (33.6)	456 (25.6)	21.5	0.173	0.031
Tracheal intubation, *n* (%)	480 (61.1)	687 (50.4)	0	0.274	0.001
First documented rhythm after hospital arrival			0	0.631	0.004
shockable	457 (47.6)	399 (22.4)	0		
non-shockable	292 (30.4)	543 (30.5)	0		
presence of pulse	211 (22.0)	836 (47.0)	0		
Time from call to hospital arrival, mins, median (IQR)	30.00 [25.00, 37.00]	30.00 [25.00, 37.00]	0.7	0.084	0.033
Adrenaline administration at in-hospital, *n* (%)	704 (73.3)	847 (47.6)	0	0.545	0.041
Coronary angiography, *n* (%)	924 (96.2)	942 (53.0)	0	1.145	0
Target temperature management, *n* (%)	614 (64.0)	748 (42.1)	0	0.449	0.071
Extracorporeal membrane oxygenation, *n* (%)	662 (69.0)	262 (14.7)	0	1.315	0.003
Cardiac cause, *n* (%)	946 (98.5)	1684 (94.7)	0	0.213	0.011

IABP, intra-aortic balloon pump; IPW, inverse probability weighting; IQR, interquartile range; SMD, standardized mean difference.

**Table 2:** Characteristics of patients in propensity-score-matched cohort.

	PS-Matched Cohort			
	With IABP	Without IABP	Missing (%)	SMD
	(N = 468)	(N = 468)		
Year, *n*(%)				
2014	39 (8.3)	40 (8.5)	0	−0.007
2015	78 (16.7)	71 (15.2)	0	
2016	75 (16.0)	80 (17.1)	0	
2017	88 (18.8)	96 (20.5)	0	
2018	98 (20.9)	85 (18.2)	0	
2019	90 (19.2)	96 (20.5)	0	
Age, years, median (IQR)	63.00 [50.00, 71.00]	64.00 [53.00, 72.00]	0	0.123
Men, *n* (%)	378 (80.8)	393 (84.0)	0	0.084
Witness status, *n* (%)	378 (80.8)	380 (81.2)	0	0.011
Bystander CPR	268 (57.3)	261 (55.8)	0	0.03
Adrenaline administration at pre-hospital, *n* (%)	121 (25.9)	134 (28.6)	0	0.062
Tracheal intubation, *n* (%)	215 (45.9)	250 (53.4)	0	0.15
First documented rhythm after hospital arrival			0	0.051
shockable	146 (31.2)	161 (34.4)	0	
non-shockable	137 (29.3)	127 (27.1)	0	
presence of pulse	185 (39.5)	180 (38.5)	0	
Time from call to hospital arrival, mins, median (IQR)	29.00 [24.00, 36.00]	30.00 [24.00, 37.00]	0	0.051
Adrenaline administration at in-hospital, *n* (%)	241 (51.5)	247 (52.8)	0	0.026
Coronary angiography, *n* (%)	432 (92.3)	437 (93.4)	0	0.041
Target temperature management, *n* (%)	238 (50.9)	287 (61.3)	0	0.212
Extracorporeal membrane oxygenation, *n* (%)	173 (37.0)	170 (36.3)	0	0.013
Cardiac cause, *n* (%)	456 (97.4)	456 (97.4)	0	0

**Table 3:** Outcomes of out-of-hospital cardiac arrest with non-traumatic origin showing spontaneous circulation return with or without IABP.

	Total		With IABP		Without IABP		Crude OR	(95% CI)	Crude OR (IPW) *	(95% CI)
All patients	(N = 2738)		(N = 960)		(N = 1778)					
CPC 1 or 2, one month after OHCA, *n* (%)	1049	(38.1)	288	(30.0)	761	(42.8)	0.57	(0.48–0.68)	1.18	(0.91–1.53)
One-month survival, *n* (%)	1487	(54.3)	480	(50.0)	1007	(56.6)	0.77	(0.65–0.90)	1.19	(0.92–1.53)
Propensity score-matched patients	(N = 936)		(N = 468)		(N = 468)					
CPC 1 or 2, one month after OHCA, *n* (%)	414	(44.2)	205	(43.8)	209	(44.7)	0.97	(0.75–1.25)	N/A	
One-month survival, *n* (%)	586	(62.6)	290	(62.0)	296	(63.3)	0.95	(0.73–1.23)	N/A	

ORs were calculated with IABP versus without IABP. * IPW analysis for ATT was performed to balance the potential confounders. IPW included the following variables: year, gender, age, witness status, bystander CPR, adrenaline administration at pre-hospital, tracheal intubation, time from call to hospital arrival, adrenaline administration at in-hospital, coronary angiography, extracorporeal membrane oxygenation, cause of arrest, target temperature management. ATT, average treatment effect among treated; CPC, cerebral performance category; CI, confidence interval; IABP, intra-aortic balloon pump; IPW, inverse probability weighting; N/A, not applicable; OR, odds ratio.

**Table 4:** Subgroup analysis of non-traumatic out-of-hospital cardiac arrest with initial shockable rhythm having a return of spontaneous circulation with or without IABP use.

	Total		With IABP		Without IABP		Crude OR	(95% CI)	Adjusted * OR	(95% CI)
Age group *										
18–64 years old	(N = 1308)		(N = 518)		(N = 790)					
CPC 1 or 2, one month after OHCA, *n* (%)	628	(48.0)	178	(34.4)	450	(57.0)	0.40	(0.31–0.50)	1.24	(0.85–1.80)
One-month survival, *n* (%)	799	(61.1)	272	(52.5)	527	(66.7)	0.55	(0.44–0.69)	1.33	(0.93–1.91)
≥65 years old	(N = 1430)		(N = 442)		(N = 988)					
CPC 1 or 2, one month after OHCA, *n* (%)	421	(29.4)	110	(24.9)	311	(31.5)	0.72	(0.56–0.93)	0.88	(0.59–1.31)
One-month survival, *n* (%)	688	(48.1)	208	(47.1)	480	(48.6)	0.94	(0.75–1.18)	1.01	(0.69–1.51)
Witness status ⁑										
Witness	(N = 2164)		(N = 764)		(N = 1400)					
CPC 1 or 2, one month after OHCA, *n* (%)	893	(41.3)	246	(32.2)	647	(46.2)	0.55	(0.46–0.66)	1.07	(0.80–1.44)
One-month survival, *n* (%)	1221	(56.4)	390	(51.1)	831	(59.4)	0.71	(0.60–0.85)	1.14	(0.85–1.54)
Without witness	(N = 574)		(N = 196)		(N = 378)					
CPC 1 or 2, one month after OHCA, *n* (%)	156	(27.2)	42	(21.4)	114	(30.2)	0.63	(0.42–0.95)	1.08	(0.53–2.21)
One-month survival, *n* (%)	266	(46.3)	90	(45.9)	176	(46.6)	0.97	(0.69–1.14)	1.54	(0.84–2.84)
First documented rhythm after arrived hospital †										
Shockable thythm	(N = 856)		(N = 457)		(N = 399)					
CPC 1 or 2, one month after OHCA, *n* (%)	260	(30.4)	135	(29.5)	125	(31.3)	0.92	(0.69–1.23)	1.45	(0.93–2.28)
One-month survival, *n* (%)	397	(46.4)	217	(47.5)	180	(45.1)	1.10	(0.84–1.44)	1.28	(0.85–1.92)
Non-shockable rhythm	(N = 835)		(N = 292)		(N = 543)					
CPC 1 or 2, one month after OHCA, *n* (%)	56	(6.7)	24	(8.2)	32	(5.9)	1.43	(0.83–2.48)	1.13	(0.54–2.36)
One-month survival, *n* (%)	196	(23.5)	93	(31.9)	103	(19.0)	1.99	(1.44–2.77)	1.49	(0.94–2.34)
Presence of pulse	(N = 1047)		(N = 211)		(N = 836)					
CPC 1 or 2, one month after OHCA, *n* (%)	733	(70.0)	129	(61.1)	604	(72.3)	0.60	(0.44–0.83)	0.78	(0.50–1.21)
One-month survival, *n* (%)	894	(85.4)	170	(80.6)	724	(86.6)	0.65	(5.30–7.89)	0.79	(0.43–1.44)
Reperfusion therapy ‡										
PCI	(N = 966)		(N = 590)		(N = 376)					
CPC 1 or 2, one month after OHCA, *n* (%)	411	(42.6)	188	(31.9)	223	(59.3)	0.32	(0.25–0.42)	0.98	(0.67–1.42)
One-month survival, *n* (%)	597	(61.8)	314	(53.2)	283	(75.3)	0.37	(0.28–0.50)	1.17	(0.79–1.73)
Non-PCI	(N = 1772)		(N = 370)		(N = 1402)					
CPC 1 or 2, one month after OHCA, *n* (%)	638	(36.0)	100	(27.0)	538	(38.4)	0.59	(0.46–0.77)	1.03	(0.69–1.52)
One-month survival, *n* (%)	890	(50.2)	166	(44.9)	724	(51.6)	0.76	(0.61–0.96)	1.10	(0.76–1.57)

CPC, cerebral performance category; CI, confidence interval; IABP, intra-aortic balloon; PCI, percutaneous coronary intervention; OR, odds ratio. ORs were calculated with IABP versus without IABP. * Adjusted for year, sex, witness status, bystander CPR, adrenaline administration at pre-hospital, tracheal intubation, first documented rhythm after hospital arrival, time from call to hospital arrival, adrenaline administration at in-hospital, extracorporeal membrane oxygenation, cause of arrest, reperfusion therapy, target temperature management. ⁑ Adjusted for year, age, sex, witness status, bystander CPR, adrenaline administration at pre-hospital, tracheal intubation, first documented rhythm after hospital arrival, time from call to hospital arrival, adrenaline administration at in-hospital, extracorporeal membrane oxygenation, cause of arrest, reperfusion therapy, target temperature management. † Adjusted for year, sex, age, witness status, bystander CPR, adrenaline administration at pre-hospital, tracheal intubation, time from call to hospital arrival, adrenaline administration at in-hospital, extracorporeal membrane oxygenation, cause of arrest, reperfusion therapy target temperature management. ‡ Adjusted for year, sex, age, witness status, bystander CPR, adrenaline administration at pre-hospital, tracheal intubation, first documented rhythm after hospital arrival adrenaline administration at in-hospital, extracorporeal membrane oxygenation, cause of arrest, target temperature management.

## Data Availability

The datasets and/or analyses in this study are not publicly available because of the lack of permission from the ethics committee.
